# Does Rapid Metabolism Ensure Negligible Risk from Bisphenol A?

**DOI:** 10.1289/ehp.0901010

**Published:** 2009-07-14

**Authors:** Gary Ginsberg, Deborah C. Rice

**Affiliations:** 1 Connecticut Department of Public Health, Hartford, Connecticut, USA; 2 Maine Center for Disease Control and Prevention, Augusta, Maine, USA

**Keywords:** β-glucuronidase, bisphenol A, endocrine disruption, fetus, glucuronidation, metabolism, neonate

## Abstract

**Background:**

Bisphenol A (BPA) risks are being evaluated by many regulatory bodies because exposure is widespread and the potential exists for toxicity at low doses.

**Objective:**

We evaluated evidence that BPA is cleared more rapidly in humans than in rats in relation to BPA risk assessment.

**Discussion:**

The European Food Safety Authority (EFSA) relied on pharmacokinetic evidence to conclude that rodent toxicity data are not directly relevant to human risk assessment. Further, the EFSA argues that rapid metabolism will result in negligible exposure during the perinatal period because of BPA glucuronidation in pregnant women or sulfation in newborns. These arguments fail to consider the deconjugation of BPA glucuronide *in utero* by β-glucuronidase, an enzyme that is present in high concentrations in placenta and various other tissues. Further, arylsulfatase C, which reactivates endogenous sulfated estrogens, develops early in life and so may deconjugate BPA sulfate in newborns. Biomonitoring studies and laboratory experiments document free BPA in rat and human maternal, placental, and fetal tissues, indicating that human BPA exposure is not negligible. The pattern of these detections is consistent with deconjugation in the placenta, resulting in fetal exposure. The tolerable daily intake set by the EFSA (0.05 mg/kg/day) is well above effect levels reported in some animal studies.

**Conclusion:**

This potential risk should not be dismissed on the basis of an uncertain pharmacokinetic argument. Rather, risk assessors need to decipher the BPA dose response and apply it to humans with comprehensive pharmacokinetic models that account for metabolite deconjugation.

Few current or past risk assessment issues are as challenging as those raised by bisphenol A (BPA). There is widespread BPA exposure to the general public, including pregnant women and infants, and the chemical is in the class of environmental hormones for which risk assessment approaches are still developing. Given that BPA is one of a large number of estrogenic chemicals to which humans are frequently exposed, this chemical represents something of a test case. Increasing the stakes is evidence for low-dose effects within the range of human exposure for end points that have implications for reproductive health and cancer. However, this evidence is in dispute, with major scientific and regulatory bodies disagreeing over BPA’s low-dose risks. Health Canada (2008) calls it a hazardous substance and has banned it from baby bottles. In contrast, the U.S. Food and Drug Administration and the European Food Safety Authority (EFSA) have found no immediate cause for concern and are not taking action to limit exposure.

Other recent reviews and commentaries have focused on the evidence of harm from low-dose exposure (Bucher 2009; Myers et al. 2009; Tyl 2008); we do not address those data here. Rather, we focus on an element that has not received critical attention: the claim raised by the EFSA that rapid metabolic clearance of BPA via first-pass glucuronide metabolism minimizes internal exposure to free BPA (EFSA 2008). We find this argument to be simplistic, ignoring several lines of evidence that internal BPA exposure can be substantial in humans despite rapid conjugation.

## The Rapid Metabolism Argument

The EFSA (2008) pointed to pharmacokinetic data in humans showing rapid BPA metabolism to the glucuronide conjugate as reason to decrease emphasis on the low-dose effects seen in rodents. Because only the parent compound binds to the estrogen receptor, conjugation is a detoxification mechanism that represents the major clearance pathway for BPA. Two studies evaluated the metabolic fate of BPA in small numbers of human volunteers ingesting low doses of deuterated BPA (d-BPA), with the labeled chemical used to increase sensitivity and distinguish administered BPA from background sources (e.g., dietary, contamination from plastics in labware) (Völkel et al. 2002, 2008). These studies failed to find detectable concentrations of free d-BPA in human plasma or urine [limit of detection (LOD) = 2.3 μg/L in plasma; see Table 1]. The kinetic profile for the conjugated metabolite (d-BPA glucuronide) showed a rapid peak followed by urinary elimination with a terminal half-life of 5.3 hr (Völkel et al. 2002). These studies have been interpreted as indicating more rapid and complete metabolic clearance of BPA in humans relative to the rat, in which circulating parent compound can be detected and the clearance of BPA glucuronide is slower (*t*_1/2_ of 20–80 hr) (EFSA 2008; Völkel et al. 2002, 2008). This was attributed to different elimination pathways in rats versus humans, given that the molecular size cutoff for urinary excretion is larger in humans (550 Da) compared with that in rats (350 Da). This results in elimination of conjugated BPA (404 Da) by biliary/fecal elimination in the rat but via urine in humans (Völkel et al. 2002). This would preclude enterohepatic recirculation in humans, in contrast to rats, in which intestinal β-glucuronidases could break down excreted conjugate and liberate BPA for systemic reabsorption. This rationale has been used to argue the irrelevance of rat data showing effects at low doses, the hypothesis being that at low doses in humans, extensive glucuronidation can essentially prevent fetal exposure to BPA.

With respect to neonatal exposure, the EFSA (2008) recognized that the human data are from adult volunteers and would not necessarily apply to neonates, who might ingest BPA via breast milk or formula. They concluded that early-life immaturity in glucuronidation capacity is likely to be augmented by sulfotransferases given that BPA is a substrate for sulfation, and there is earlier ontogeny of sulfotransferases compared with UDP-glucuronosyltransferases. This was demonstrated for the therapeutic drug acetaminophen, which undergoes a shift in metabolic clearance from primarily sulfation to primarily glucuronidation with increasing postnatal age (Allegaert et al. 2005; Levy et al. 1975; Miller et al. 1976). Therefore, the EFSA (2008) concluded that between sulfation and glucuronidation, sufficient BPA conjugation capacity exists in neonates to prevent exposure to the parent compound.

Based on these arguments, the EFSA maintains the tolerable daily intake (TDI) of BPA for the European Union at 0.05 mg/kg/day (EFSA 2008), an exposure rate that is 200 times greater than that associated with adverse effects in some rodent studies (Vandenberg et al. 2007).

## Why Rapid Metabolism Is Not the End of the Story

Although cross-species pharmacokinetic differences exist, there is still ample opportunity for human exposure to free BPA. That is because β-glucuronidases exist not only in the intestines but also throughout the body, including the placenta and fetal liver. This creates the potential for local activation of the conjugated form back to free BPA in numerous tissues. This is analogous to the situation for endogenous estrogens, which are transported as sulfate conjugates and can be cleaved to active hormone by tissue sulfatases (Iwamori 2005). β-Glucuronidase activity is widely distributed throughout mammalian tissues and is present in both the endoplasmic reticulum and lysosomes (Paigen 1989; Sperker et al. 1997). Although its primary physiologic role is the degradation of proteoglycans, the enzyme is also able to deconjugate a variety of xenobiotic substrates that have already undergone phase II glucuronidation. This can result in conjugation–deconjugation cycling that does not involve enterohepatic recirculation, as documented for the drug diflunisal. Treatment of rats with a specific β-glucuronidase inhibitor decreased metabolic clearance of diflunisal by 54% in experiments in which the bile duct was cannulated to prevent enterohepatic recirculation (Brunelle and Verbeeck 1997). Deconjugation of a variety of other xenobiotic metabolites has been documented in human liver preparations, including acetaminophen (Bohnenstengel et al. 1999) and the aromatic amines benzidine and 4-aminobiphenyl (Zenser et al. 1999). β-Glucuronidase protein levels and enzyme activity were readily detectable and varied widely in human liver and kidney samples taken from 30 and 18 individuals, respectively (Sperker et al. 1997).

Additional evidence also argues against a simplistic approach. A reanalysis of the National Health and Nutrition Examination Survey (NHANES) BPA urinary biomonitoring results from 1,469 adult participants suggested a longer than expected half-life of BPA (Stahlhut et al. 2009) based on modeling of the fasting time (before sample draw) relative to urinary concentration and the assumption that most BPA exposure comes from the diet. The stable level of urinary BPA may have been due to nondietary BPA exposures, tissue accumulation and storage of free BPA, and/or conjugation–deconjugation cycling of BPA involving β-glucuronidase. It is noteworthy that whereas the terminal half-life is reported to be only 5.3 hr in humans after d-BPA administration (Völkel et al. 2002), the time course shows no additional d-BPA glucuronide removal beyond 20 hr (additional data from Völkel et al. 2002 presented in Teeguarden et al. 2005). This is consistent with delayed excretion due to long-term tissue storage and/or conjugation–deconjugation cycling. The terminal half-life in cynomolgus monkeys dosed orally with ^14^C-BPA was 9.7 hr (Kurebayashi et al. 2002). Thus, the cross-species difference in terminal half-life of BPA glucuronide may not be as large as stated (EFSA 2008). Finally, there are uncertainties when comparing half-life across studies involving administered oral doses that were 100–1,000 times higher in the rat study (Domoradzki et al. 2003; Pottenger et al. 2000) than in the human study (Völkel et al. 2002). Although glucuronidation is considered a high-capacity pathway, one cannot rule out the possibility that the rat to human difference in half-life was affected by the difference in administered dose.

Developmental studies suggest rapid ontogeny of β-glucuronidase, because it is detected prenatally in liver, kidney, and lung in a variety of laboratory species, with activity particularly high in placenta (Lucier et al. 1977). Human placenta also has considerable β-glucuronidase activity, and this enzyme is critical for proper *in utero* development (Collier et al. 2009; Paigen 1989; Sperker et al. 1997). An inherited deficiency leads to hydrops fetalis, a birth defect related to improper fetal breakdown of mucopolysaccharides and water accumulation. Because glucuronidation capacity is immature in early life, the net balance tends to be toward deconjugation in the animal models and tissues studied (Lucier et al. 1977). This makes BPA deconjugation a potentially important pharmacokinetic factor during the perinatal period.

Thus, it is apparent that one has to consider the potential for β-glucuronidase–mediated deconjugation of BPA glucuronide in placental and fetal tissues. Even though glucuronidation may be rapid, the reported terminal half-life of circulating BPA glucuronide, 5.3 hr, affords ample opportunity for transport to placenta and deconjugation back to BPA. This and the fact that the fetus itself contains β-glucuronidase increase the chances for substantive fetal exposure to free BPA. By focusing on rapid conjugation and not considering sites of deconjugation other than the intestine (enterohepatic recirculation), the EFSA has not considered the implications of BPA glucuronide deconjugation in placental and fetal tissues. This issue may be a key determinant of cross-species extrapolation of BPA internal dose, but it has yet to be addressed in BPA pharmacokinetic models (Edginton and Ritter 2009; Teeguarden et al. 2005) or regulatory determinations.

## What Is the Meaning of Free BPA Detection in Human Samples?

The Völkel et al. (2002, 2008) human dosing studies failed to detect free BPA (Table 1) and so were interpreted by the EFSA (2008) as supportive of rapid metabolic clearance and negligible exposure in adults as well as the fetus. However, numerous other studies have detected free BPA in both humans and rats, either associated with general background exposure or in experiments where rodents were dosed with BPA (Table 1). Free BPA was detected by a variety of methods and was found not only in adult blood but also in placental and fetal samples. Dekant and Volkel (2008) argued that the detection of free BPA in such studies may result from background contamination from labware and indoor dust. Further, some free BPA may be formed by cleavage from the glucuronide present in the sample when readying the sample for analysis (Dekant and Volkel 2008). However, many of the cited studies were aware of these potential artifacts and took steps to prevent false-positive detection of free BPA. Further, our review of the free BPA data derived from these studies suggests that this result is not artifactual. Quite the contrary, these data suggest patterns of occurrence that have important implications for BPA risk assessment.

**Figure 1:**
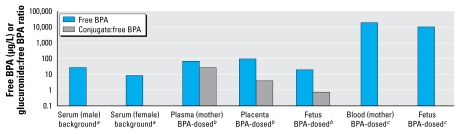
Free BPA and ratio of conjugate to free BPA in rats from background exposure or after BPA dosing. ^***a***^Data from Takeuchi et al. (2004). ^***b***^Data from Domoradzki et al. (2003). ^***c***^Data from Takahashi and Oishi (2000).

**Figure 2:**
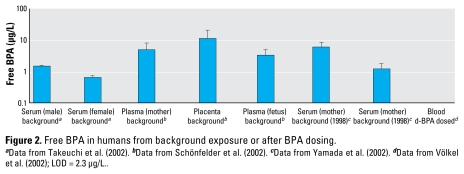
Free BPA in humans from background exposure or after BPA dosing. ^***a***^Data from Takeuchi et al. (2002). ^***b***^Data from Schönfelder et al. (2002). ^***c***^Data from Yamada et al. (2002). ^***d***^Data from Völkel et al. (2002); LOD = 2.3 μg/L..

Figure 1 includes studies in which free BPA was detected in rats dosed during pregnancy, demonstrating maternal serum BPA greater than fetal BPA in two studies (Domoradski et al. 2003; Takahashi and Oishi 2000). In a study in which placental BPA was also measured, the placenta had a higher BPA concentration than did the maternal or fetal compartments. This is consistent with human free BPA data collected in a biomonitoring study of pregnant women exposed to background sources of BPA (Schönfelder et al. 2002). Once again, placenta had the highest concentration, followed by maternal and fetal compartments (Figure 2). These trends have a plausible biological basis in that placenta has extensive β-glucuronidase activity (see above) and so may be an important site of metabolite deconjugation and resultant fetal exposure. Although peak fetal concentrations of free BPA were less than maternal concentrations in both rats and humans, a more detailed time course in rats indicated that cumulative free BPA exposure was actually greatest in the fetus [fetal area under the curve was 73% greater than maternal (Takahashi and Oishi 2000)]. This may reflect ongoing deconjugation in placenta and fetus that prevents free BPA from declining as rapidly as in the maternal system. Also of note in this study was the finding of much higher concentrations of free BPA in liver and kidney compared with blood, again suggesting the importance of local tissue deconjugation and/or binding in determining free BPA dose. In this study, Takahashi and Oishi (2000) used a very large dose (1 g/kg), so their results, although consistent with the others cited, should be repeated at more relevant doses.

Additional evidence of BPA deconjugation during gestation comes from rat data showing that the ratio of glucuronide to free BPA varies across maternal, placental, and fetal compartments (Figure 1). Although Domoradzki et al. (2003) reported no selective tissue affinity for BPA or its metabolite, their data show a continuous decrease in glucuronide to free BPA ratio across these compartments, suggesting a greater role for deconjugation in the placenta and fetus than in maternal blood.

The biological plausibility of the free BPA data is also supported by the difference between sexes in both rats and humans (Figures 1 and 2). Male rats exhibited greater free BPA than did females, consistent with decreased expression of the main BPA-glucuronidating enzyme [UDP-glucuronosyltransferase 2B1 (UGT2B1)] in male rat liver (Takeuchi et al. 2004). The fact that this was also seen in humans (Figure 2) suggests that BPA metabolic fate is under hormonal control in both species. The sex differential appears to exist very early in human development, because free BPA was greater in male than female fetuses of women receiving BPA exposure from background sources (Schönfelder et al. 2002).

The risk implications of free BPA detections need to be explored based upon dose–response assessment and suitable physiologically based pharmacokinetic (PBPK) modeling that can relate internal dose of free BPA to adverse effect. The existing PBPK models (Edginton and Ritter 2009; Teeguarden et al. 2005) have not considered the influence of local deconjugation reactions. In the only attempt to simulate free BPA concentrations, Teeguarden et al. (2005) were not able to reproduce the free BPA results for rat plasma at the later time points (4 and 8 hr after dosing) even though their model included enterohepatic recirculation and plasma protein binding. There is a clear need to improve modeling efforts with respect to free BPA in maternal, fetal, and neonatal tissues across species, with metabolite deconjugation a potentially important element. Better calibration of the models against the database of free BPA detection (Table 1) should be part of this effort. This may be facilitated by *in vitro* studies that evaluate the conjugation–deconjugation activity of placenta and other human and rodent tissues. Such *in vitro* data along with additional human volunteer studies evaluating BPA concentration after controlled exposures (e.g., Carwile et al. 2009) will inform the degree of variability in human BPA pharmacokinetics. This is particularly uncertain given the small numbers of adult subjects that were involved in the detailed pharmacokinetic studies thus far reported (Völkel et al. 2002, 2008; Table 1). A PBPK model parameterized with empirical conjugation and deconjugation rate constants and calibrated for free BPA is needed to relate the dose response for toxic effects found in rodents to humans.

## What about BPA in Neonates?

The EFSA also considers dietary BPA exposures in neonates to be insignificant because of rapid metabolism, in this case not because of glucuronidation but because of sulfation. Based on analogy with acetaminophen, BPA may be conjugated with sulfate rather than glucuronide in neonates because of the earlier development of sulfotransferases. However, sulfation does not end the biological activity of endogenous hormones, so there is no reason to believe it will do so for sulfated BPA. Sulfated estrogens, mainly in the form of estrone sulfate, have a long half-life in blood, where their concentration is much higher than the active hormone (Nakamura et al. 2005). These conjugates act as a circulating reservoir of inactive hormone that can be deconjugated in local tissues by arylsulfatase C, a widely expressed microsomal enzyme that is especially prevalent in estrogen-responsive tissues (Reed et al. 2005; Tobacman et al. 2002). Given that sulfotransferases also exist in these tissues, the balance between conjugation and deconjugation at a particular life stage and in a specific tissue is a key determinant of local estrogen dose. This has not been studied for BPA and is a critical data need for developing improved PBPK models for the postnatal period. However, it is reasonable to assume that the BPA sulfate conjugate would be subject to deconjugation in a manner similar to endogenous sulfated estrogens. This is pertinent to the postnatal period as arylsulfatase C activity develops *in utero* and is readily detectable in human neonatal liver (Richard et al. 2001). Thus, sulfation of BPA in neonates does not guarantee negligible internal dose as assumed by the EFSA.

Another consideration is that this argument is based on analogy with acetaminophen. However, it is uncertain whether the sulfotransferases present in neonates will be as efficient in conjugating BPA as they are for acetaminophen. This represents another key data gap. Finally, genetic polymorphism in major sulfotransferases such as SULT1A1*2 can decrease conjugating activity 2- to 10-fold (Hildebrandt et al. 2007; Nagar et al. 2006; Ohtake et al. 2006). This can be an important source of interindividual variability in neonatal BPA conjugation that is not considered in the EFSA assessment.

## Summary

Free BPA concentrations have been detected in a wide range of both human and rodent studies and likely reflect the *in vivo* condition rather than artifact. This provides evidence of exposure to free BPA in human adults and fetuses despite rapid first-pass glucuronidation. Deconjugation at local tissue sites by the action of β-glucuronidase and arylsulfatase C provides a plausible mechanism. The EFSA’s review of the pharmacokinetic evidence concludes that cross-species differences in BPA glucuronide fate (enterohepatic recirculation in rats; urinary excretion in humans) makes low-dose studies in rodents less relevant for human risk assessment (EFSA 2008). The EFSA also believes that on the basis of rapid BPA metabolism and excretion in humans, fetal and neonatal exposure is negligible. However, detection of free BPA in human and rodent placenta and fetus is contrary to that opinion and argues for placing importance on deconjugation reactions in future risk assessments.

The EFSA TDI of 0.05 mg/kg/day (EFSA 2008) is orders of magnitude greater than the dose found to produce effects in some rodent studies. Pharmacokinetic differences across species would have to be enormous to justify acceptance of a TDI that far above possible effect levels. The points raised above demonstrate the uncertainty in the pharmacokinetic argument for maintaining the current EFSA TDI. Efforts should be placed on deciphering the dose–response relationship in rodent studies and applying it to human risk assessment based upon PBPK models that account for metabolite deconjugation in conjunction with other pharmacokinetic factors. Such PBPK models do not currently exist, and the existing models (Edginton and Ritter 2009; Teeguarden et al. 2005) cannot fully simulate the human and rodent data. Therefore, as an interim measure, one may choose to directly apply the rodent dose–response relationship to humans and seek additional mechanistic or epidemiologic data to refine the human risk assessment.

## Figures and Tables

**Table 1:** Studies evaluating free BPA in biological fluids.

Study	Exposure	Analytical method	Results	Comments
Domoradzki et al. 2003	Pregnant rats gavaged with ^14^C BPA, 10 mg/kg, on GD11, GD13, or GD16	Radiochemical HPLC: LOD ~ 35 μg/L; tests run to ensure that procedures did not cleave BPA glucuronide to BPA	Free BPA not detected in many samples early in gestation; GD16 free BPA detectable in all tissues: placenta > maternal plasma > fetus; ratio of free/conjugated: fetus > placenta > maternal plasma	Although fetus had 3.6-fold less free BPA than maternal plasma, free/conjugated BPA ratio was much greater in fetus; may reflect altered conjugation/deconjugation balance in fetal compartment; data from early period before enterohepatic circulation created a secondary *C*_max_

Ikezuki et al. 2002	Humans (mother/fetus) background exposure (*n* = 32–38)	ELISA: details not given, but accuracy checked against standard HPLC method	Free BPA detected in maternal serum, ovarian follicular fluid, and cord blood at similar levels; amniotic fluid was 5 times higher in early but not late pregnancy	Higher free BPA in amniotic fluid early in pregnancy may be related to changing composition: coming from maternal plasma early vs. fetal urine late in gestation; free BPA is in maternal plasma but unlikely in urine

Schönfelder et al. 2002	Humans (mother/fetus) background exposure (*n* = 37)	GC-MS: LOD = 0.01 μg/L LOQ = 0.1 μg/L	Free BPA detected in placenta > maternal plasma > fetal plasma; fetal > maternal in 14 of 37 samples, with male fetus > female fetus in these cases	Methods avoided BPA leaching from labware into sample; differences across tissues and sexes suggest free BPA is biologically based rather than from background contamination

Takahashi and Oishi 2000	Pregnant rats dosed on GD18 with 1 g/kg gavage	HPLC: LOD = 5 μg/L	Free BPA detected in maternal tissues > fetal tissue = maternal blood; fetal *t*_1/2_ 3 times greater than maternal *t*_1/2_	High-dose rat study has limited relevance, but it demonstrates distribution to fetal compartment; enterohepatic recirculation likely affects *t*_1/2_ in both fetus and mother but does not explain longer *t*_1/2_ in fetus

Takeuchi et al. 2004	Rats background exposure (*n* = 10/sex)	HPLC: details not clear but appear to involve standard solvent extraction	Free BPA detected in males > females	Sex differential corresponds to lower BPA conjugating capacity in male liver microsomes and lower expression of UGT2B1

Takeuchi and Tsutsumi 2002	Humans: background exposure (*n* = 11 men, 14 women)	ELISA: accuracy checked against HPLC method	Free BPA detected in males > females	Free BPA correlated with serum testosterone in both men and women, suggesting androgen effect on BPA fate

Tan and Mohd 2003	Humans: background exposure (*n* = 180 females)	GC-MS: LOD = 0.05 μg/L	Free BPA detected in cord blood in 88% of samples	Demonstrated potential utility of biomonitoring free BPA and other alkylphenols in cord blood

Völkel et al. 2002	Humans: dosed orally with 5 mg d-BPA (*n* = 4)	LC-MS: LOD_plasma_ = 2.3 μg/L; LOD_urine_ = 1.4 μg/L	No detection of free BPA even though d-BPA glucuronide was high (160 μg/L blood); d-BPA glucuronide, *t*_1/2_ = 5.3 hr	Lack of free BPA attributed to “practically complete” first-pass metabolism and lack of enterohepatic circulation; detection of free BPA in background samples attributed to plastic contamination

Völkel et al. 2008	Humans: background exposure (*n* = 287)	LC-MS: LOD_urine_ = 0.3 μg/L; LOQ_urine_ = 1.25–5 μg/L	10% of urine samples with low detection (> LOD, < LOQ)	Free BPA in urine attributed to contamination from house dust or plastics because BPA was also found in blanks; d-BPA dosing did not find free BPA in urine

Völkel et al. 2008	Human: dosed orally with 5 mg d-BPA (*n* = 1)	HPLC-MS: LOD_urine_ for d-BPA was not stated	Free d-BPA not detected in any urine samples from this subject	Results used to assert that if free BPA is detected in urine, it is from contaminants and not free BPA

Yamada et al. 2002	Humans: background exposure (*n* = 200 women)	ELISA: LOD = 0.2 μg/L; method previously validated against HPLC method	Second trimester serum samples in Japanese women showed steady decline in free BPA from 1989 to 1998	No clear explanation of decreasing BPA in serum, but results were significant (*p* < 0.001); free BPA lower in amniotic fluid than maternal serum

Abbreviations: *C*_max_, maximum concentration; ELISA, enzyme-linked immunosorbent assay; GC, gas chromatography; GD, gestation day; HPLC, high-performance liquid chromatography; LC, liquid chromatography; LOQ, limit of quantification; MS, mass spectrometry.
